# Updated treatment guidelines for patellar instability: “un menu à la carte”

**DOI:** 10.1186/s40634-021-00430-2

**Published:** 2021-11-26

**Authors:** David H. Dejour, Guillaume Mesnard, Edoardo Giovannetti de Sanctis

**Affiliations:** 1 Clinique de la Sauvegarde, Ramsay Santé, 8, Avenue Ben Gourion, 69009 Lyon, France; 2 Lyon Ortho Clinic, 29 Av. des Sources, 69009 Lyon, France

## Introduction

Patellofemoral disorders could be named the worse pathology to treat for an orthopaedic surgeon. The high variability in terms of clinical figures, psychologic consequences on sometimes very young patients with parents who often feel guilty of the situation, the poor and pretty unspecific clinical exam and the necessity of a perfect technical imaging mixing plain radiographs and slice imaging with a high level of knowledge to interpret them. This makes the final diagnosis and the treatment algorithm difficult, whereas taking all these data step by step could lead to a more simple and secure care for the patient.

The goal of this article is to clarify, to simplify the way to overlook the patient in the light of experience and objective data coming from literature.

## Patellar instability: definition

Patellofemoral instability is a multifactorial disease including a spectrum of different conditions.

Patellar dislocations occur more frequently in the second decade of life, mainly in female adolescent patients. The incidence of patellar instability varies between 5.8/100,000 and 29/100,000 in the 10-17 year old age group [36].

The Patellofemoral joint stability depends on both bone morphology and soft tissue restraints, and it has a role of fundamental importance for proper functioning of the knee extensor mechanism.

The groove lateral facet is higher, larger and more protuberant anteriorly than the medial one.

The patellar articular side has similar and congruent characteristics. The importance of the trochlear lateral facet in resisting lateral forces is rational and widely accepted.

Furthermore, the lateral retinaculum is stronger and wider in relation with the medial side.

A mechanical imbalance, often associated with anatomic abnormalities, with laterally overcoming the medially directed forces, may lead to dislocation.

H. Dejour and the school of Lyon [14] classified patients having patellofemoral (PF) pathology associated with instability in three groups: Objective Patellar Instability/ Objective Patellar Dislocation (OPI/OPD), Potential Patellar Instability/ Potential Patellar Dislocation (PPI/PPD) and Painful patellar syndrome (PPS) using clinical history and an objective description with statistical threshold for anatomical abnormalities (Table 1).

**Table 1:** Classification according to H. Dejour et al.

Groups	*Knee Pain*	*Anatomic Risk factors*	*Documented Dislocation*
**OPI/OPD**	X	X	X
**PPI/PPD**	X	X	
**PPS**	X		

OPI or OPD group includes patients with two characteristics: both a history of at least one documented patellar dislocation, defined as a complete displacement of the patella with respect at least one anatomical risk factor.

The cases of pure traumatic patellar dislocation are rare and therefore excluded from this classification. These patients, due to their normal patellofemoral anatomy, usually have a patella which is difficult to be relocated within the groove after the acute episode.

The dislocation has to be objectively documented; mostly children and adolescent may confuse episodes of patellar subluxation as true complete dislocation totally different from the feeling of instability related to the quadriceps inhibition.

OPI/OPD group may be further classified, based on the number and type of the dislocation in recurrent (more than 3 episodes), habitual (during each early knee flexion) and permanent (always dislocated throughout the whole range of motion) patellar dislocation.

More frequently patients within this group have a complete displacement with no pain reducing spontaneously, due to the presence of anatomical abnormalities influencing the normal stability of the patella.

PPI or PPD group includes patients with at least one anatomic instability factor complaining of knee pain but without reporting any documented patellar dislocations. Maltracking and subluxations (defined as partial loss of contact) might be found in the affected or more commonly in the contralateral knee.

PPS group includes patients complaining of knee pain but without having any anatomic instability factor or reporting any documented dislocation/subluxation. Actually, large part of this group does not belong to spectrum of disorders termed patellar instability.

## Clinical presentation and examination

### Common symptoms

Anterior knee pain, subjective feeling of unstable knee, and locking or catching are frequent clinical symptoms developing in patients with patellar instability. During the clinical observation the physician should figure out whether the patella is centered within the groove or if it is permanently subluxated/dislocated.

### Main signs and tests

These signs are fundamental, influencing significantly the diagnosis and treatment.

The Apprehension test, the Patellar Tilt test, the quadrant test, the J sign and/or abnormal patellar tracking are the clinical aspects, preferred by the authors, to evaluate the patients.

The Apprehension test is a physical finding in which forced lateral displacement of the patella produces anxiety and resistance in patients with a history of lateral patellar instability.

The Patellar Tilt test evaluates the amount of clinical patellar inclination, this test is performed with the knee in full extension.

The medial tilt test identifies the reducibility of the patellar tilt and the tightness of the lateral retinaculum.

The quadrant test is done in extension and flexion to evaluate the MPFL competency.

Maltracking refers to the dynamic malalignment of the patella within the trochlear groove occurring during active or passive range of motion of the knee.

The J-sign is the clinical sign of the disengagement in extension of the patella from the trochlea; it is an active sign leaded by the quadriceps contraction, it means more often a mechanical or functional patella alta, a short or convex trochlea.

The abnormal patellar tracking is only visible in high-grade trochlear dysplasia. The patella could dislocate or reduce during active or passive ROM.

A dislocation in flexion means a shorter proximal or distal extensor mechanism, whereas a dislocation in extension means only a high trochlear dysplasia with a normal length of the quadriceps.

## Anatomic risk factors and radiologic evaluation

This is the key to set the treatment decision. A deep knowledge of the different anatomic abnormalities, leading to PF instability, is necessary to choose the right treatment for each patient.

In 1987 H. Dejour et al. [14] described the four major anatomical factors leading to patellar dislocation: trochlear dysplasia (TD), patella alta (PA), excessive TT-TG distance and patellar tilt.

In the last decade, several authors have confirmed and highlighted the importance of those risk factors, unless the patellar tilt angle whose relevance has slightly decreased to become a consequence of the others.

True sagittal view, axial view at 30° of knee flexion and anteroposterior view have to be evaluated and correctly done by radiologist for a correct treatment. The lateral view has to be performed superimposing the two posterior femoral condyles in a monopodal weight-bearing position with 20° of flexion.

CT and MRI show perfectly the global shape of the trochlea and are able to quantify the axial malalignment (TT-TG), the patellar tilt and some rotational deformities.

### Trochlear dysplasia

Trochlear dysplasia is the main and the first risk factor most frequently associated with patellar instability, diagnosed in up to 96% of patients with OPI [14].

It refers to a pathologic alteration in the shape of the femoral trochlea (flat or shallow groove with or without an associated supratrochlear prominence), which always has a genetic origin.

**Fig. 1:**
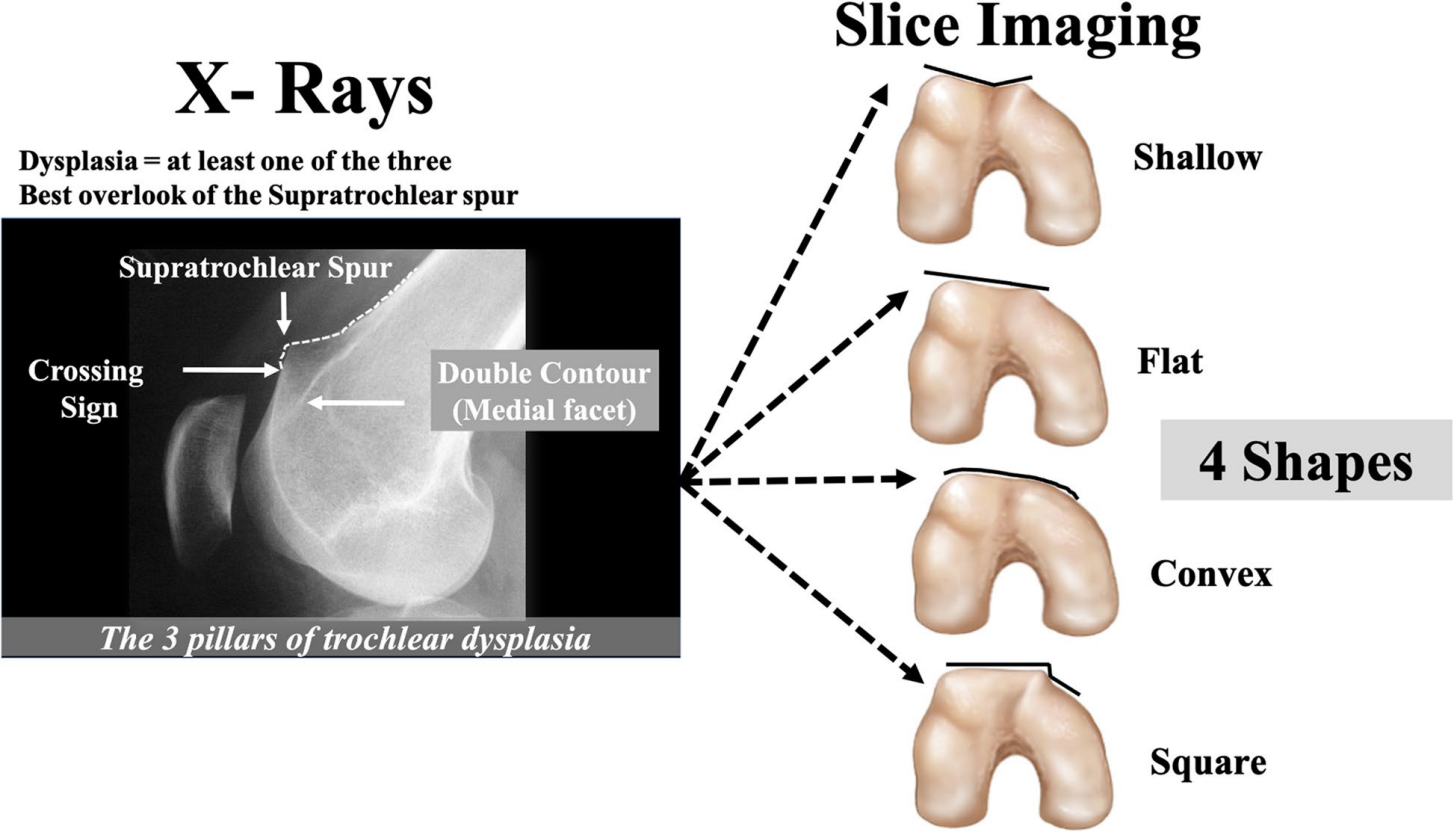
Two Imagings are mandatory to screen the trochlear dysplasia. X-Ray shows the 3 pillars of trochlear dysplasia. Slice imaging gives the shape of the trochlea but not the supratrochlear spur

On the radiograph sagittal view the trochlear dysplasia is defined by three pillars: the crossing sign, the supratrochlear spur and the double-contour sign. The slice imaging is helpful in defining the shape of the trochlea. The combination of both imagings is mandatory to have a clear definition of the trochlear dysplasia (Fig. 1).

The crossing sign, first described by H. Dejour et al. [14], is defined as the point where the trochlear sulcus radiographic line links the projection of the anterior femoral condyles. It represents macroscopically the exact position where the sulcus and anterior femoral condyle have the same AP height, indicating that a segment of the trochlea has become a flat. The lower the ‘crossing sign’, the higher the grade of trochlear dysplasia.

**Fig. 2:**
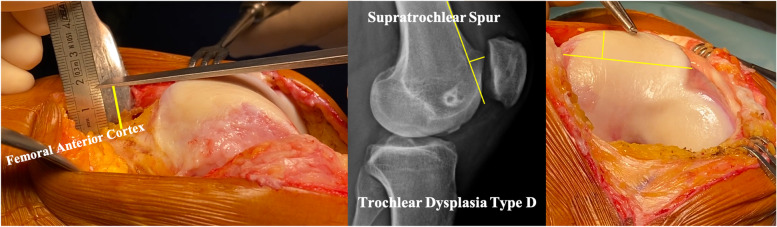
Definition of the supratrochlear spur

The supratrochlear spur is a protuberance (bump or prominence) on the superolateral part of the trochlea with a functional effect, during the trochlear engagement, similar to the ski ramp (Fig. 2).

The double-contour sign is seen radiologically as a double line at the anterior aspect of the condyles. It represents medial hypoplastic facet with an inferior AP height compared to the sulcus and lateral condyle; it has to end below the crossing sign.

Axial views obtained with the knee flexed 30° might be used to evaluate the shape of the trochlea, measure the sulcus angle and also evaluate the patella dysplasia classified according to Wiberg.

The sulcus angle (defined by Brattström [7]) is calculated by drawing two lines, from the deepest point of the groove, towards the most superior point of each condyle. The mean normal sulcus angle value, evaluated on radiographs, is 142° ± 0.5 [7]. In trochlear dysplasia the sulcus angle is increased or unmeasurable.

At our institution we do not commonly use the sulcus angle to quantify the trochlear dysplasia. Axial radiographs are used only to evaluate the presence of condylar or patellar fractures. To analyze the shape of trochlear in the axial plane, we prefer the use of slice imaging like CT scan or MRI.

**Fig. 3:**
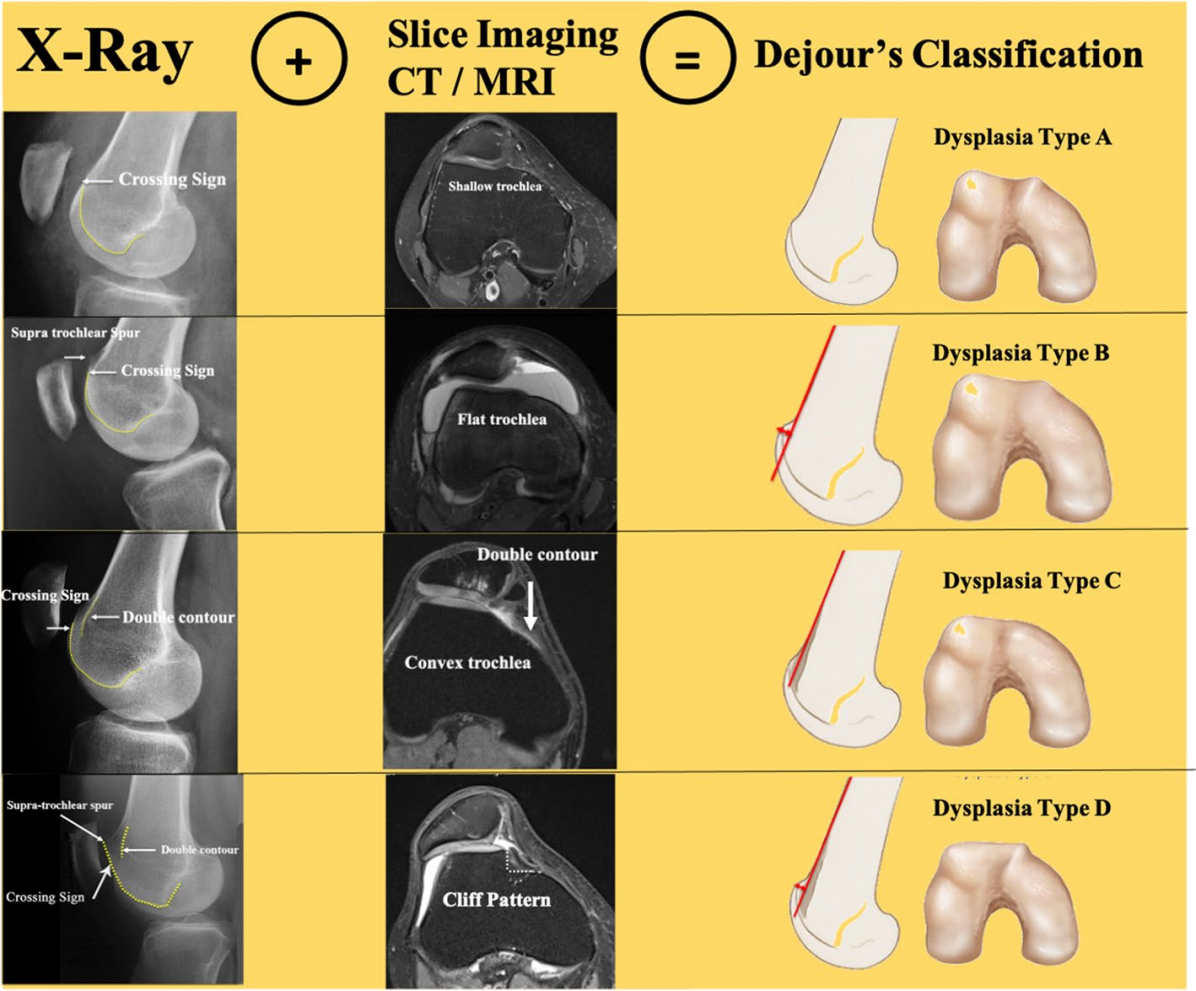
Classification of the 4 types of trochlear dysplasia according to Dejour et al. [12] matching X-rays and Slice imaging

Basically, by cross-checking the radiographic sagittal view and the cross-sectional images, trochlear dysplasia might be classified, according to Dejour et al. [12], as shown in Table 2 (Fig. 3).

**Table 2:** The Dejour’s classification of trochlear dysplasia

Type	*Sagittal View*	*Axial images*
**A (54%)**	CS	Shallower trochlea
**B (17%)**	CS and SS	Flat or convex
**C (9%)**	CS and DC	Convex lateral facet and hypoplastic medial facet
**D (11%)**	CS, SS, DC	A prominent and convex lateral facet with a vertical connection (squared) to an hypoplastic medial facet almost absent (cliff pattern).

Cross-checking the radiographic sagittal view and the cross-sectional images is fundamental to include a patient within one of those trochlear types

*CS* Crossing sign, *SS* Supratrochlear spur, *DC* Double contour

### Patella alta

Patella alta is the second major risk factor which could lead to patellar dislocation alone.

The intrinsic function of the patella is to increase the moment arm and therefore the effective extension force of the quadriceps muscle. The position of the patella related to the trochlea will have a huge effect on the patellofemoral stability but also on potential pain.

In normal knees, patellar engagement with the trochlea occurs at around 20° of flexion and the.

PF total contact area increases from extension to flexion and reaches a maximum at 90°.

Patella Alta refers to an abnormal and more proximal position of the patella in relation to the femur.

When the patella is alta it leads to a delay of the patellar engagement within the groove during the early phase of flexion. An increase in the “free” range of motion, with the patella out of the restraining bony supports, would facilitate lateral dislocation, due to the usual prevalence of the lateral structures with respect to the medial ones.

Patella Alta increases the quadriceps moment arm, resulting in greater compression forces and decreases the PF contact area between 0° and 60° of flexion; those two anomalies lead to a higher risk of cartilage degeneration and subsequent pain [20].

Dejour et al. [13] showed that 24% and 90% of patients with Objective Patellar Instability (OPI) had respectively patella Alta and patellar Tilt.

Several methods have been described to quantify patellar height on sagittal radiographs [5, 19, 21]. Our preferred index to evaluate this aspect, is the Caton-Deschamps index (CDI), due to the simplicity of the measurement and as it is not affected by tibial tuberosities abnormalities [8, 9] and it is easy with that to find out the exact amount of distalization needed.

Furthermore, an International Patellofemoral Study Group consensus established the CDI as the preferred method for measuring patellar height [22].

It is the ratio between the distance from the patellar inferior pole to antero-superior tibial plateau (AT) and the length of the patellar articular surface (AP).

A ratio > 1.2 and < 0.6 are referred to as respectively patella alta and patella infera.

Agreement between patellar height measurements from radiographs, MRI and CT remains unclear. Variability in knee positioning quadriceps contraction and imaging modality may modify CDI measurement.

The MRI is performed with the patient in a supine position with extended knees.

Caton-Deschamps index on MRI is calculated with the sagittal slice showing the patella with the greatest length in the plan of ACL.

Yue et al. [37] showed greater CDI values on MRI than radiographs, with a difference varying from 0.17 to 0.18.

Paul et al. stated that the CDI has strong agreement between radiographic and magnetic resonance imaging. The average Caton–Deschamps index was 1.23 ± 0.18 on radiograph and 1.26 ± 0.18 on magnetic resonance imaging, with a mean difference of − 0.03 ± 0.15.

Apart from the CDI, the Lyon protocol considers the use of another Index, measuring the patellar height and its functional engagement with the trochlea.

Sagittal engagement index is measured as the ratio between the articular cartilage of the patella and the trochlear cartilage length measured on two different MRI slices.

**Fig. 4:**
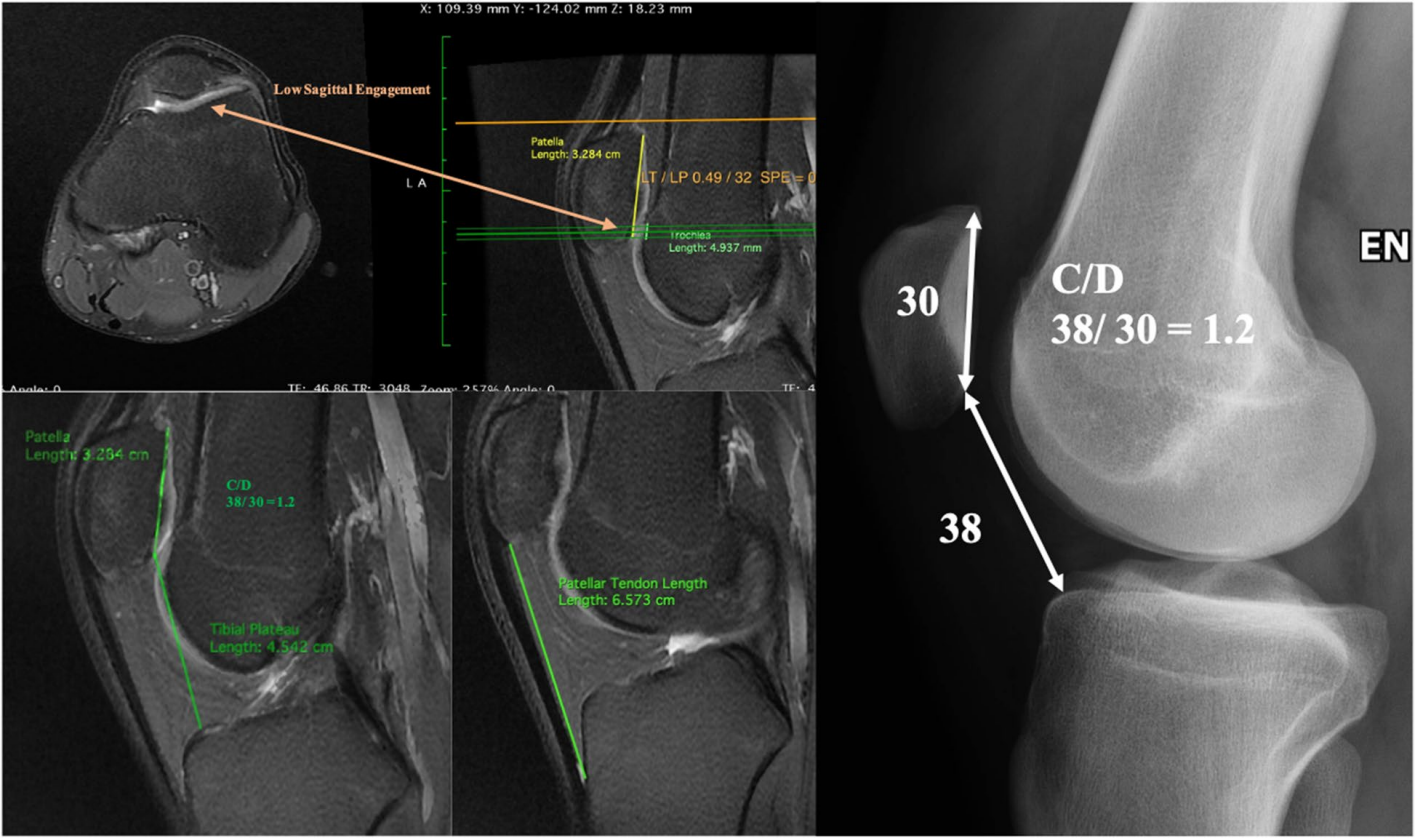
Patella Alta quantification: X-ray with the Caton-Deschamps index, MRI with the Sagittal Patellar Engagement index, notice the patellar tendon length

Dejour et al. [11] introduces a new method to measure the Sagittal Patellofemoral Engagement (SPE) Index with the use of MRI. It might serve as a supplementary tool to the existing methods of evaluating patellar height, and may help to better identify the cases where inadequate engagement is recorded despite the absence of patella alta measured on x-rays (Fig. 4).

They showed that a SPE < 0.45 in a patient with patellar dislocation might be considered patella alta with an insufficient functional sagittal PF engagement.

Two distinctive sagittal cuts are selected. On a first sagittal cut, showing the longest patellar articular cartilage, patellar length (PL) line is drawn, measuring the entire length of the patellar articular cartilage. On a second sagittal cut, showing the trochlear cartilage extending more proximally, PL and a line parallel to it (TL – Trochlear Length) with the same starting height are drawn. The TL line reaches the most proximal articular trochlear cartilage. The SPE is therefore calculated as the ratio between TL and PL.

The previously published method [4] of patellotrochlear index on a single image slice, does not allow its use in cases of dislocated patella.

It is always interesting to look at indirect signs of patella alta on slice imaging (CT scan or MRI): the highest axial images show the patella but not the trochlea, which is visible only once the patella has disappeared.

Another way to evaluate patella alta is by evaluating axial CT images of the patellofemoral joint with and without quadriceps contraction. The quadriceps muscle contraction often creates a more pathological displacement of the patella with a clinical maltracking or a J sign.

### TT-TG distance

TT-TG (tibial tuberosity-trochlear groove) distance is the third risk factor for patellar instability but differently from trochlear dysplasia and patella alta it is not able to lead individually to to patellar dislocation.

The TT-TG distance represents the radiographic measurement of the lateral quadriceps vector acting on the patella.

This value is modified by both femoral/tibial rotation and varus/valgus knee coronal alignment, altering the Q angle, formed by the lines of pull of the quadriceps and patellar tendon intersecting at the center of the patella. In order to measure this angle accurately the patella should be centered on the trochlea.

An excessive femoral anteversion, tibial external rotation, subtalar joint pronation or genu valgus and genu recurvatum are the secondary anatomic instability factors altering the coronal, clinically evaluated, Q angle and therefore the axial, radiologically measured, TT-TG distance.

A greater femoral anteversion, by rotating the distal femoral epiphyses internally, increases the lateral vector leading to both a higher risk of lateral compartment degeneration and patellar dislocation [33].

An increased TT-TG distance might lead to patellofemoral disorders.

The TT-TG distance is assessed according to Lyon Protocol [14]. The first cranial axial image depicting a complete cartilaginous trochlea is used to draw a line (trochlear line), perpendicular to the posterior condylar tangent, through the deepest point of the groove. A second line, parallel to the trochlear line, is drawn through the most anterior portion of the tibial tubercle. The distance between those 2 lines representes the TT-TG distance.

Although it is a very controversial value due to the low reproducibility, it is the best index produced so far to measure the Q angle. The TT-PCL value, which has been proposed recently [31], has not shown any superiority to the TT-TG in differentiating patients with patellofemoral instability [6], but remain a good alternative to quantify the axial malalignment. However, the key point is to have measures helping the physician in evaluating the patient and planning the surgery preoperatively and that could be used to assess the surgical result.

Traditionally a pathological TT-TG distance, evaluated on CT Scan, has a cut off value of 20 mm.

Recently, several authors have used magnetic resonance imaging (MRI) to calculate the TT–TG distance proposing a cut-off value of around 13 mm [34, 35].

### Patellar tilt

During the 80’s the patellar tilt used to be reported as a PF instability risk factor but later it has proved to be only a consequence of the other three risk factors described above. Patellar tilt refers to the inclination of the patella in relation to the posterior bicondylar line. An abnormal tilt value is the result of many factors, including both trochlear/patellar morphology and medial/lateral restraints tightness imbalance. Debated is whether the Patellar tilt is a sensitive marker for patellar instability as it may occur without patellar subluxation/dislocation [16].

Patellar tilt is traditionally measured on CT views and it is described by the angle formed by a line tangent to the posterior femoral condyles and a line passing through the transverse axis of the patella.

In the study by Dejour et al. [14], 83% of patients with at least one episode of patellar dislocation had a patellar tilt value greater than 20°.

In the Lyon CT Scan protocol the tilt is measured in extension with and without quadriceps contraction; the difference between those two values is the expression of the disengagement of the patella from the trochlea, describing radiologically the clinically evaluated J-Sign.

### Soft tissue status in patellar dislocation

Stabilizers of the PF joint might be divided into two groups: static-passive and active-dynamic. Imbalance among those would predispose to patellar malalignment and instability. Passive stabilizers include both patellofemoral and patellotibial ligaments.

The MPFL (Medial Patello-Femoral Ligament) acts as a static restraint to lateral translation of the patella. Desio et al. [15] reported that 60% of the force directed medially and therefore restraining the lateral dislocation is produced by the MPFL. It is fundamental to understand that the MPFL rupture is the consequence of the lateral patellar dislocation and never the cause and that dislocations do not occur without causing the MPFL rupture.

The MPFL reconstruction is therefore not a realignment procedure but should be considered as a check-rein for the patella.

MPTL has shown more recently to have an important role in patellar stability and it is responsible of most of the patellar bony avulsions [38].

The iliotibial band, have some attachments to the patella, contributing to the lateral retinaculum.

The development of an abnormal bone morphology during growth leading to a chronic patellar tilting and shifting contributes to a tight and thick lateral reticulum.

We should not forget one of most important dynamic stabilizers: the VMO (Vastus Medialis Obliquus) formed by two parts, the vertical and the oblique ones. Usually in chronic patellar instability, the vertical part which has higher insertion on the patella is associated with an hypoplastic or absent oblique part, leading to a biomechanically non efficient action of this muscle [17, 18].

## Treatment Lyon’s algorithm

The algorithm needs to be characterized by objective, reliable and measurable data. This is the necessary condition to be successful in the highest number of patients.

The knowledge of patient’s history and the clinical exam is a necessary but not sufficient condition. The final decision is made on a careful analysis of both X-rays and slices imaging.

**Fig. 5:**
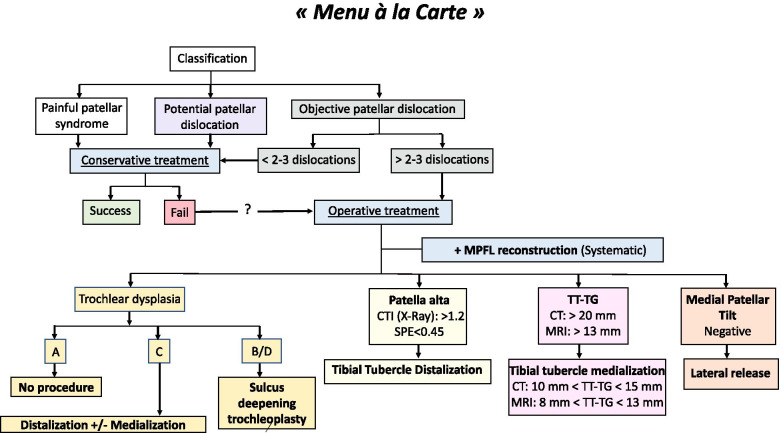
The updated treatment algorithm for patellar instability. Each abnormality has to be evaluated and surgically corrected when indicated (“menu à la carte”)

Patients have to be divided in categories based on their anatomic features and a “menu à la carte” should be applied to correct each abnormality performing the appropriate surgical procedure (Fig. 5) [26, 30].

### First dislocation management

**Fig. 6:**
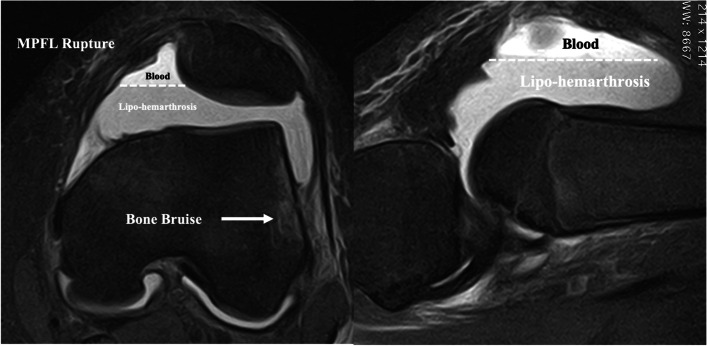
First time patellar dislocation with Lipohemarthrosis, MPFL rupture and Bone bruise. Remember to search for osteochondral fractures!

After acute first-time patellar dislocations, conservative treatment is indicated. Coronal, sagittal and axial views are performed to first assess the presence of patellar or condylar fractures and then to explore anatomic risk factors. MRI images are indicated in emergency only in skeletally immature patients ruling out osteochondral fractures, which impose an immediate surgery (Fig. 6).

No immobilization is required in order to avoid knee stiffness. A light brace and/or crutches might be used to decrease pain during the first week.

In case of a severe and painful lipohemarthrosis, an arthrocentesis might be considered to aspirate the intra-articular liquid and reduce the pain caused by the increased pressure.

The rehabilitation program aims to restore complete range of motion, strengthen the quadriceps and stretch the knee lateral compartment soft tissues and finally guide the medial side healing.

Several authors have proposed surgical treatment after a first-time patellar dislocation, consisting of acute repair or reconstruction of the torn MPFL. Debated is whether the surgery leads to better clinical outcomes and decreased risk of re-dislocation than conservative management [1, 27]. Currently expert consensus suggests a conservative treatment after first time dislocation.

The patient is visited at 45 days to evaluate the anatomic risk factors, give a prognosis on the recurrence rate and explain the patient what could be the future.

### Objective patellar dislocation

#### Trochlear dysplasia

Trochlear dysplasia type A does not require a specific surgical treatment to modify the shape of the groove.

Trochlear dysplasia types B and D fit the best with sulcus deepening trochleoplasty, due to the trochlear prominence (supratrochlear spur) [2].

This procedure’s indications are precise, i.e. recurrent patellar dislocations with both high-grade trochlear dysplasia (B or D), and patellar maltracking. The contraindications are: established patello-femoral osteoarthritis, open growth plates and a painful knee with no dislocations.

**Fig. 7:**
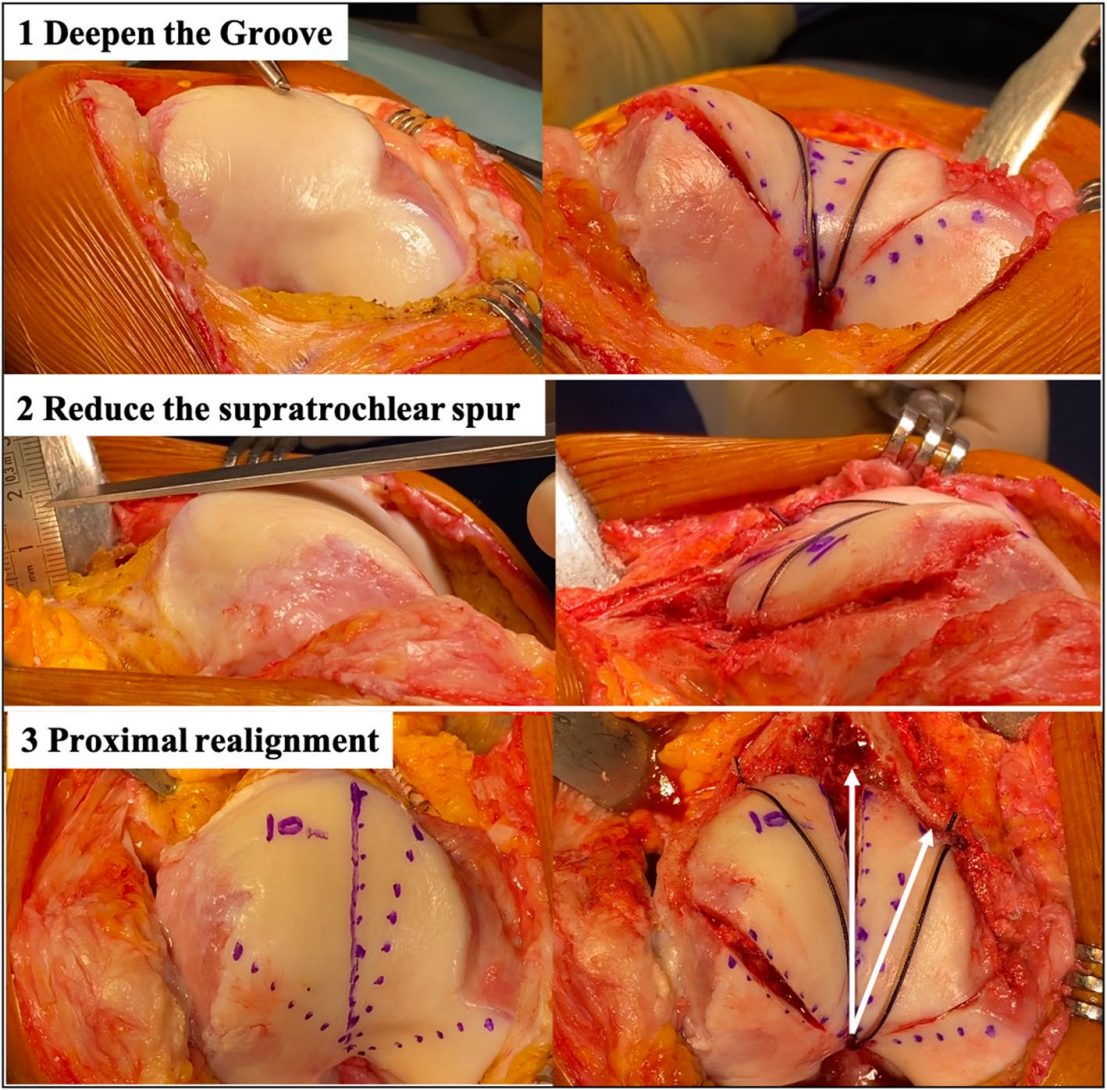
Three goals of the deepening trochleoplasty: 1) Deepen the trochlea. 2) Reduce the supratrochlear spur and make the trochlea flushes with the femoral anterior cortex. 3) Proximal realignment with the new groove aligned with the anatomical femoral axis

Sulcus deepening trochleoplasty has three functions: it modifies the trochlear shape with a central groove and oblique medial and lateral facets; it decreases the patellofemoral joint reaction force by reducing the supratrochlear prominence (spur); and reduces the TT-TG value by the groove repositioning (proximal realignment), often without further need of tibial tubercle medialization (Fig. 7).

This procedure has shown good clinical outcomes with improved PF stability [10, 23, 26].

Still debated is the proper treatment for trochlear dysplasia type C. Many options have been proposed: tibial tubercle distalization of 5 mm combined with a lateral retinaculum release or a lateral facet elevating procedure [3] which is the preferred option.

#### Patella alta

In case of a Caton-Deschamps Index and/or a low Sagittal Patellofemoral Engagement (SPE) Index respectively greater and smaller than 1.2 and 0.45, a tibial tubercle distalization is performed. The goal is to obtain a CDI of 1. Therefore, a patient with a CDI of 34/26 would need a distalization of 8 mm.

This procedure enables the patella to engage the trochlear groove in extension.

#### TT-TG and secondary anatomic factors

Tibial tubercle osteotomy with a medialization decreases the extensor mechanism valgus force and is indicated in case of an excessive TT-TG distance. Based on whether the measurement is taken on CT scan or MRI, the cut-off value would be respectively 20 mm and 13 mm.

This procedure’s goal is to change the TT-TG distance, within a range of 10 to 15 mm and 8 to 13 mm respectively if measured on CT scan or MRI.

For example, the amount of medial tubercle medialization in a patient with a CT scan measured TT-TG distance of 23 mm, would be 10 mm in order to obtain a post-operative value of 13 mm.

The five secondary anatomic instability factors previously described, altering the axial alignment are considered of secondary surgical importance as no pathologic cut-off values with an associated surgical treatment algorithm have been detected. In carefully selected patients, proximal femoral and tibial derotation osteotomy might be performed in case of respectively excessive femoral anteversion and tibial external rotation. Femoral or tibial osteotomy might correct a severe valgus knee.

#### MPFL and patellar tilt

The surgical planning should include systematically a MPFL reconstruction to improve the subjective result by the check-rein effect. Several techniques, grafts and fixation methods have been proposed so far, without proving one of those to be superior [28, 29].

To reduce the high rate of failure rate [32], an isolated MPFL reconstruction should not be considered as a realignment procedure. Therefore, the perfect indication is a low-grade trochlear dysplasia (type A), a normal or subnormal TT-TG and no patella alta.

Lateral release might be performed only in cases of clinical lateral tightness (Negative medial patellar tilt test) [24, 25]. The patella tilt angle value has lost its previous role within the treatment algorithm. The cut-off value, calculated on CT scan of 20° is not used anymore.

This procedure might be performed open (e.g. once associated with sulcus deepening trochleoplasty) or arthroscopically. Finally the VMO plasty is not performed anymore.

## Updates summary

This updated treatment algorithm reflects the experience gained during the last decades while treating this specific and controversial knee pathology.

It confirms that the scientific approach proposed in 1987 was successful. Originally the Lyon’s team defined and described the instability factors and proposed statistical thresholds within a “menu à la carte” algorithm. Years after years the knowledge became the property of the orthopedic community around the world and brought some confirmations, validations but also some new factors and items within the algorithm. This is a perfect example of a worldwide collaboration.

The conclusion highlights the major impact of intrinsic instability factors. The first, in order of importance, intrinsic factor is trochlear dysplasia which needs to be screened by radiographs and slice imaging. The second is patella alta which needs to have a correlation between the bone evaluation on radiographs and the cartilage assessment on MRI by looking at the sagittal patello-trochlear engagement, which is a huge added value in surgical indication. The third intrinsic factor is the axial alignment, quantified by the TT-TG distance, taking into account the different thresholds of CT Scan and MRI. In patellofemoral disorders always evaluate the global aspect of the patient by looking at the secondary factors. The role of tilt and the way of calculating it has changed slightly within the algorithm and it is referred to as a consequence of the other factors. The eventual presence of VMO dysplasia keeps on being assessed but does not correspond to any surgical procedure anymore.

The surgical options have changed so far and the techniques became more and more reliable, e.g.the MPFL reconstruction definitely killed the so called “Insall plasty” .

The key to treat patients with patellar dislocation is to make the right diagnosis, which is validated by at least one of the instability factors and then apply the algorithm, consisting in correcting one by one them. This, will prevent misdiagnosis, overdiagnosis and limits the iatrogenic surgery.

The “menu à la carte” have helped us to successfully and better treat patellar dislocations that once seemed impossible. However, we could clearly state that not all the answers have been found but we are moving closer to the goal.

## Data Availability

Not applicable.
